# ENHANCING DEMENTIA-FRIENDLY COMMUNITIES IN TOKYO, JAPAN THROUGH COMMUNITY-BASED PARTICIPATORY RESEARCH

**DOI:** 10.1093/geroni/igae098.3342

**Published:** 2024-12-31

**Authors:** Mika Sugiyama, Hiroki Inagaki, Kae Ito, Shuichi Awata

**Affiliations:** Tokyo Metropolitan Institute for Geriatrics and Gerontology, Tokyo, Japan; Tokyo Metropolitan Institute for Geriatrics and Gerontology, Tokyo, Japan; Tokyo Metropolitan Institute for Geriatrics and Gerontology, Tokyo, Japan; Tokyo Metropolitan Institute for Geriatrics and Gerontology, Tokyo, Japan

## Abstract

In Japan, grappling with the challenges of an increasingly aging population, the Basic Law on Dementia was enacted in June 2023 to foster the development of dementia-friendly communities (DFCs) nationwide. Over the past decade, starting from 2013, a community-based participatory research (CBPR) initiative has been underway in Chiyoda Ward, Tokyo, aimed at establishing a comprehensive dementia support system. Monthly team meetings have served as forums for discussing institutional progress and addressing challenges, with efforts toward DFCs guided by the PDCA cycle. In partnership with Chiyoda Ward, a longitudinal mail survey was conducted to assess the needs of approximately 10,000 older adults. For non-respondents, visiting nurses conducted home visits to provide early-stage dementia support. Additionally, a novel system was implemented to extend “early support” and “watchful waiting support by visiting nurses” to individuals at high risk of dementia who did not engage with public consultation services. Chiyoda Ward has actively organized “dementia cafes” and support meetings for affected individuals, along with training “dementia supporters” for community volunteering. Additionally, the introduction of the “Chiyoda Dementia Support Company/University Certification System” in 2022 has acknowledged outstanding efforts, resulting in the certification of six entities. Notably, there has been a positive trend in the proportion of elders feeling confident about living safely within their community despite dementia, as indicated by the mail survey increasing from 28% in 2016 to 35% in 2021. As a result of CBPR efforts, significant progress has been made in the social implementation of DFC in Chiyoda Ward.

